# Identifying Neurocognitive Decline at 36 Months among HIV-Positive Participants in the CHARTER Cohort Using Group-Based Trajectory Analysis

**DOI:** 10.1371/journal.pone.0155766

**Published:** 2016-05-18

**Authors:** Marie-Josée Brouillette, Tracy Yuen, Lesley K. Fellows, Lucette A. Cysique, Robert K. Heaton, Nancy E. Mayo

**Affiliations:** 1 Department of Psychiatry, McGill University, McGill University Health Centre, Montreal, Canada; 2 Institute of Health Policy, Management and Evaluation, University of Toronto, Toronto, Canada; 3 Child Health Evaluative Sciences, Hospital for Sick Children, Toronto, Canada; 4 Department of Neurology & Neurosurgery, McGill University, Montreal Neurological Institute, Montreal, Canada; 5 University of New South Wales, St. Vincent's Hospital Sydney, Neuroscience Research Australia, Sydney, Australia; 6 HIV Neurobehavioural Research Centre, University of California San Diego, San Diego, California, United States Of America; 7 Division of Clinical Epidemiology, McGill University Health Centre, Montreal, Canada; University Of São Paulo, BRAZIL

## Abstract

**Introduction:**

While HIV-associated neurocognitive impairment remains common despite the widespread use of combined antiretroviral therapy (cART), there have been relatively few studies investigating the trajectories of neurocognitive change in longitudinal NeuroAIDS studies.

**Objective:**

To estimate the magnitude and pattern of neurocognitive change over the first 3 years of follow-up using Group-Based Trajectory Analysis (GBTA) applied to participants in the longitudinal arm of the CHARTER cohort.

**Method:**

The study population consisted of 701 CHARTER participants who underwent neuropsychological (NP) testing on at least 2 occasions. Raw test scores on 15 NP measures were modeled using GBTA. Each trajectory was categorized as stable, improved or declined, according to two different criteria for change (whether the magnitude of the estimated change at 36 months differed ≥ 0.5 standard deviations from baseline value or changed by > the standard error of measurement estimated at times 1 and 2). Individuals who declined on one or more NP measures were categorized as decliners.

**Results:**

Overall, 111 individuals (15.8%) declined on at least one NP test over 36 months, with the vast majority showing decline on a single NP test (93/111-83.8%). The posterior probability of group assignment was high in most participants (71%) after only 2 sessions, and in the overwhelming majority of those with 3+ sessions. Heterogeneity of trajectories was the norm rather than the exception. Individuals who declined had, on average, worse baseline NP performance on every test, were older, had a longer duration of HIV infection and more follow-up sessions.

**Conclusion:**

The present study identified heterogeneous trajectories over 3 years across 15 NP raw test scores using GBTA. Cognitive decline was observed in only a small subset of this study cohort. Decliners had demographics and HIV characteristics that have been previously associated with cognitive decline, suggesting clinical validity for the method.

## Introduction

In settings where combination antiretroviral therapy (cART) is widely available, the burden of neurocognitive complications has shifted from HIV-Associated Dementia to milder forms of HIV-related neurocognitive impairment [1,2]. These milder forms of impairment are nonetheless associated with decreased ability to function in everyday life[3], making them a focus of concern. Detecting *decline* in cognitive function may be particularly important clinically, as it suggests an active, potentially reversible process that requires further investigation and perhaps changes in management.

Cognitive decline is typically identified with repeat neuropsychological (NP) testing. From a statistical standpoint, modeling longitudinal data presents several challenges. The pattern of longitudinal change may not be monotonic, may not be the same for each person, and the probability of change may depend on the starting point. Attrition from the cohort that is not appropriately accounted for will bias the estimates and any modeling of the data needs to consider the structure of the correlation matrix over time. Analyses that simply calculate differences within individuals over time may not adequately address these challenges, potentially yielding biased estimates of change. Finally, NP data in particular pose an additional challenge because they are conventionally interpreted in relation to the performance of a normative sample, with norms required both for cross-sectional performance and change over time. What constitutes appropriate norms is a matter of debate: while norms for cognitive change in HIV+ individuals have been proposed[4], their generalizability needs to be further demonstrated. Addressing these issues is a critical first step to allow the study of the mechanisms underlying cognitive decline in HIV.

New analytical approaches to longitudinal data are now available to address the complexity of longitudinal data. The strength of these modern approaches is that change can be observed, parameterized, and then interpreted without the need for an external benchmark (i.e. norms) and without assuming that change is linear. This paper illustrates the use of one such method, Group-Based Trajectory Analysis (GBTA). GBTA assumes the population is made up of a mix of people with different longitudinal trajectories and uses a semi-parametric approach to group people with a similar pattern together[5,6]. Initially developed for applications in the social sciences, this method has now been applied in the health field, including in the analysis of cognitive change over time[7–9]. For example, Xie et al. [9] applied this method to analyze change in Mini-Mental Status Examination scores over 3.5 years in 187 geriatric patients with mild cognitive impairment. They identified 5 distinct groups of people with varying rates of decline. One such group was of particular interest: it included 6 individuals in whom the decline was much sharper than the rest of the sample. Importantly, the unusual trajectory followed by these individuals was masked when data were analyzed using more standard repeated measures analyses, such as linear mixed-models, which explain variation around a common group mean. This ability of GBTA to delineate distinct patterns of change over time is one of its major strengths compared to existing approaches. Finally, GBTA is also flexible towards missing information as even people who miss visits or are lost to follow-up can be assigned to a trajectory based on their available data.

In this paper, we will illustrate the pattern of change observed in HIV+ individuals participating in the CNS HIV Anti-Retroviral Therapy Effect Research (CHARTER) study. The specific aims are to characterize how scores on NP tests evolve over time to identify individuals who likely show cognitive decline, and to determine the extent to which this decline relates to selected demographics and HIV-related variables known to predict decline.

## Methods

### Sample Population

The study population consisted of 701 participants in the longitudinal arm of the CHARTER study who underwent NP testing on at least 2 occasions. Participants were recruited between September 2003 and August 2007 in six university centers across the United States. Inclusion and exclusion criteria were broad such that the sample population would be reflective of all HIV patients presenting at HIV clinics[1]. Written informed consent was obtained from all study participants, and the secondary analysis of the data was approved by the Psychiatry/Psychology Research Ethics Board (REB) of the McGill University Health Centre (13-214-PSY). The study protocol has been fully described elsewhere [1]

### Measures

At time of recruitment and semi-annually thereafter, all participants completed a battery of 15 NP tests covering 7 cognitive domains known to be commonly affected in people with HIV [1].

### Statistical Methods

GBTA was used to identify distinct trajectory groups that best fit the data, described by polynomial regressions. Each NP test was analyzed with separate models. Once the best-fitting trajectories were identified, individuals were assigned a probability of belonging to each trajectory (termed “posterior probability”). The largest posterior probability defined trajectory group membership for each person. Fit of the model was considered very good when the average probability of group membership is ≥ 80%. Raw test scores of each NP test were modeled using GBTA [5,6] to identify groups of study participants with similar changes in NP test scores over the course of the follow-up period. Models with different numbers of trajectory groups and parameterization of time (e.g. linear, quadratic or cubic) were compared using fit statistics and posterior group probabilities.

### Definition of change

GBTA describes longitudinal change but cannot be used to determine the relevance of the observed patterns. There is no gold standard in this setting, i.e. the minimal clinically important difference for each NP test has not been established. One potential solution is using norms for change, but as for norms more generally, their use rests on the assumption that the available normative data apply to the sample under study, which can be difficult to verify. Here, we adopted a different approach to determine whether deterioration in raw scores represented a meaningful decline. We tested two definitions of change relying on established conventions in longitudinal clinical research. In the absence of an established criterion for minimal clinically important difference, change is often defined based on estimates derived from the observed distribution of the sample; most commonly a change equal to or greater than 0.5 standard deviation (SD) from the baseline score is considered meaningful [10], so we adopted this threshold here as our first definition of change.

However, this definition does not address the concern that an artificial improvement in score upon repeated testing, i.e. a practice effect (PE), can be observed on NP tests. The complexity of accounting for this phenomenon is magnified by the fact that both the presence and magnitude of the PE varies between individuals and tests. We considered this potential artifact as contributing to measurement error in the current sample. Robust data on the Standard Error of Measurement (SEM) associated with each NP test, which is typically determined with test-retest experiments specifically designed for this purpose, are not available. Therefore, we simulated a test-retest experiment in our large sample. We selected the first two test sessions that occurred 6 months apart among individuals who were aviremic at both sessions: PE is usually more important between the first and the second session [11], and six months is not considered long enough to show clinical-biological change among aviremic individuals in the absence of a major health event[4], thus variation in NP scores could reasonably be attributed to SEM. SEM was estimated from the sample data using the standard formula of Standard Error of Measurement = (SD of the sample differences between the scores at t1 and t2) / square root of 2 [12]. Change greater than this ad hoc SEM estimate was our second definition of change. Values representing change using both definitions are shown in Table 1.

**Table 1:** Distribution derived values to assign change: 0.5 standard deviation (SD) and Standard Error of Measurement (SEM).

TEST	0.5 SD	SEM
TMT-A	5.9	7.09
Digit Symbol	9.2	5.91
Symbol Search	4.5	3.62
TMT-B	24.1	24.10
Wisconsin Card Sorting Test	4.5	6.70
Category Fluency	2.7	3.14
Letter Fluency	6.2	5.05
PASAT	6.2	5.33
L-N Sequencing	1.5	1.51
BVMT-learning	3.4	3.66
HVLT- learning	2.7	2.66
BVMT-Recall	1.3	1.49
HVLT- recall	1.4	1.59
GP Dominant	9.8	9.10
GP Non Dominant	12.3	14.90

Each trajectory from the 15 models was then categorized as stable, improved or declined based on the predicted NP score at 36 months, the median time of follow-up, using the estimated regression parameters (i.e. intercept and beta-coefficients) of each trajectory. Accordingly, trajectories that predicted a decline in score ≥ 0.5 SD or > SEM within 36 months were classified as declined, while trajectories which predicted improvement in test scores of the same magnitude over 36 months were classified as improved; the remaining trajectories that did not meet the threshold for meaningful change were labeled as stable.

Each participant was then assigned to one specific trajectory for each test, based on the largest posterior probability of group membership. Participants who were assigned to one or more trajectories categorized as declined were considered decliners. The odds of being classified as decliner or non-decliner (including stable and improving) were estimated and the two groups were compared in terms of baseline scores on each NP test and on a composite measure of NP performance (the Global Deficit Score- GDS[13]) used in the research setting to detect HIV-associated cognitive impairment, and on selected demographic characteristics using t-tests for continuous variables and chi-square tests for categorical variables.

Analyses were conducted using SAS Version 9.3 [14].

## Results

Among the CHARTER cohort, 701 participants had at least one follow-up session of NP testing and are included in the analysis. Graphs of the 15 trajectory models are presented in Figs 1–15.

**Fig 1:**
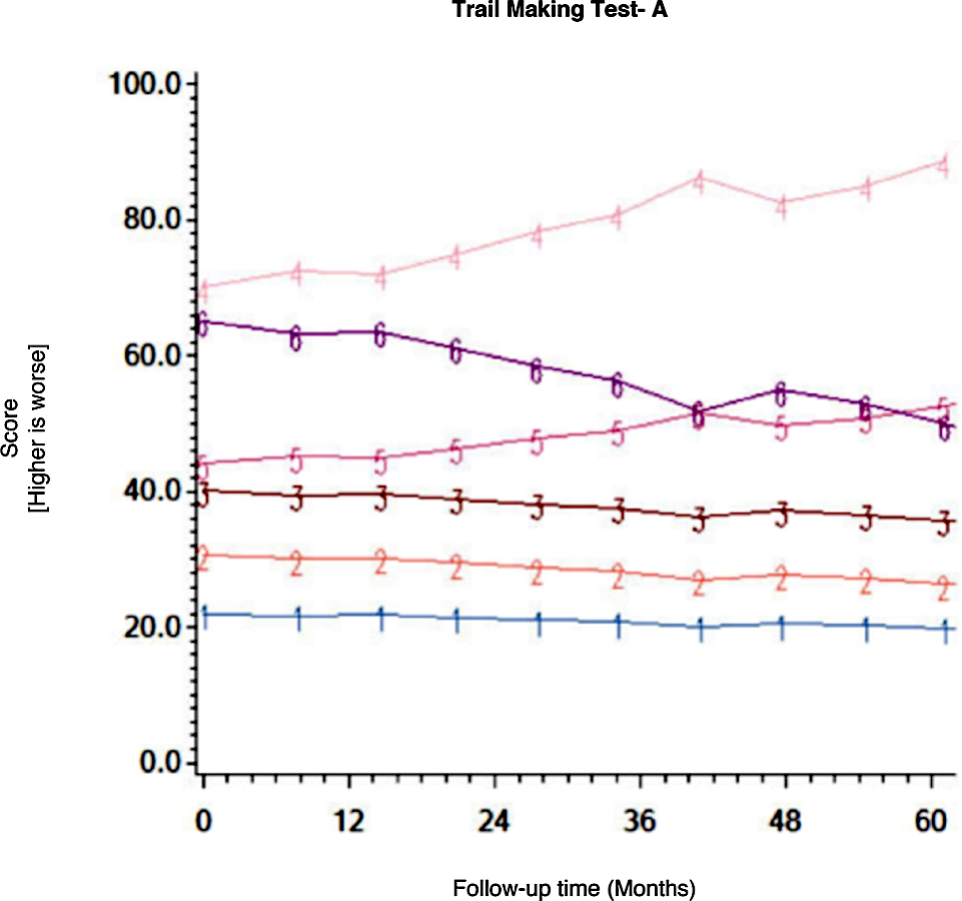
Results of group-based trajectory analysis for Trail Making Test A.

**Fig 2:**
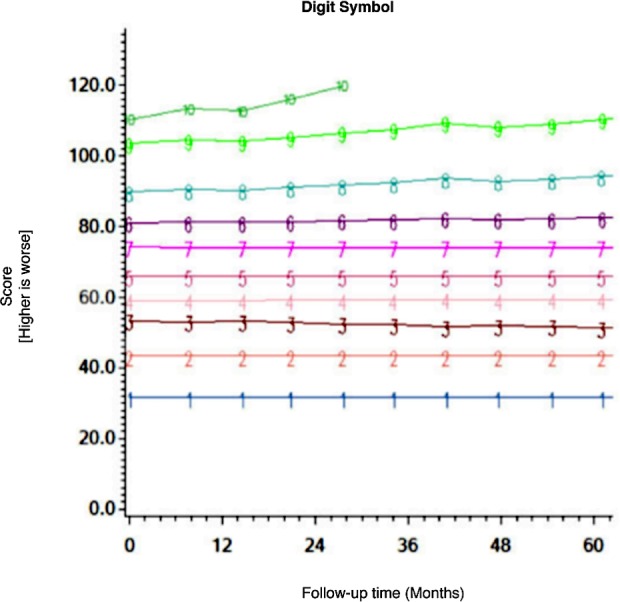
Results of group-based trajectory analysis for Digit Symbol.

**Fig 3:**
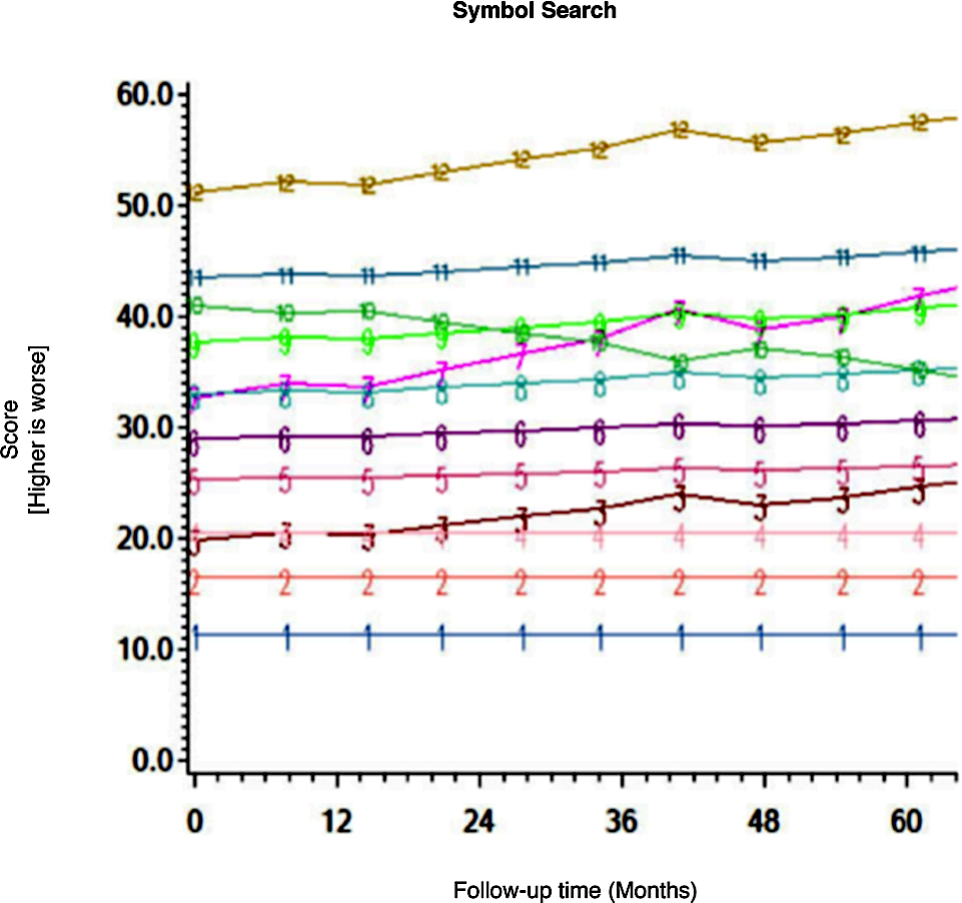
Results of group-based trajectory analysis for Symbol Search.

**Fig 4:**
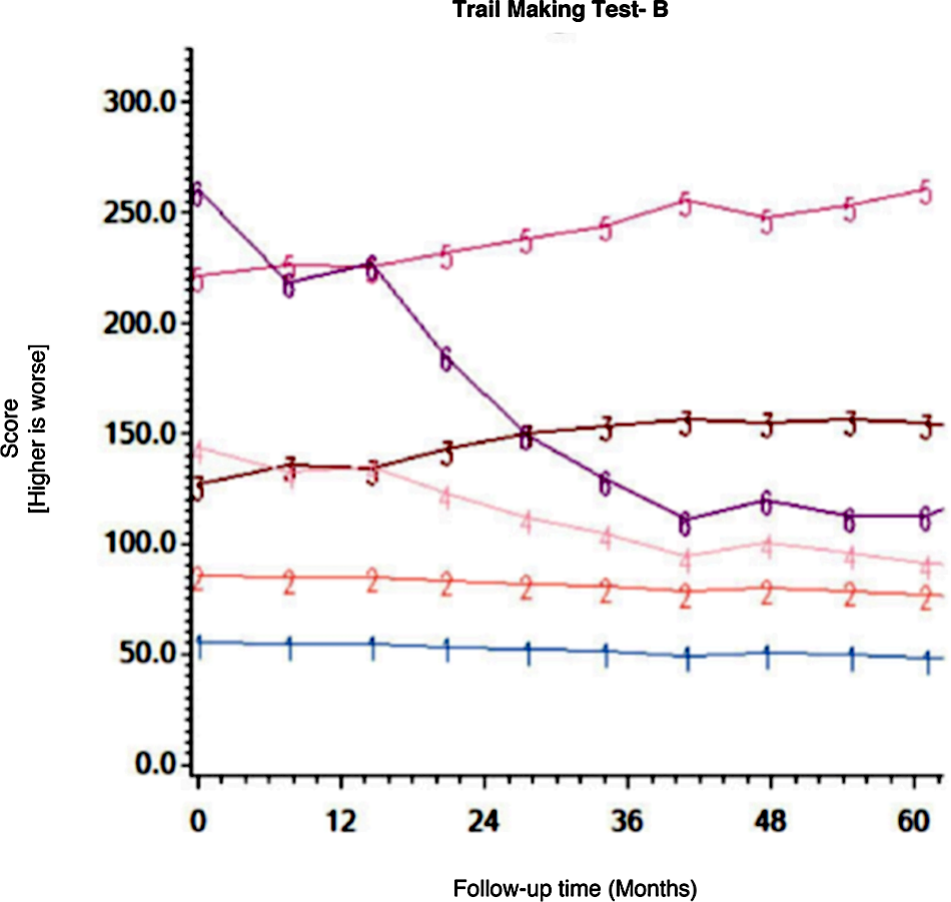
Results of group-based trajectory analysis for Trail Making Test B.

**Fig 5:**
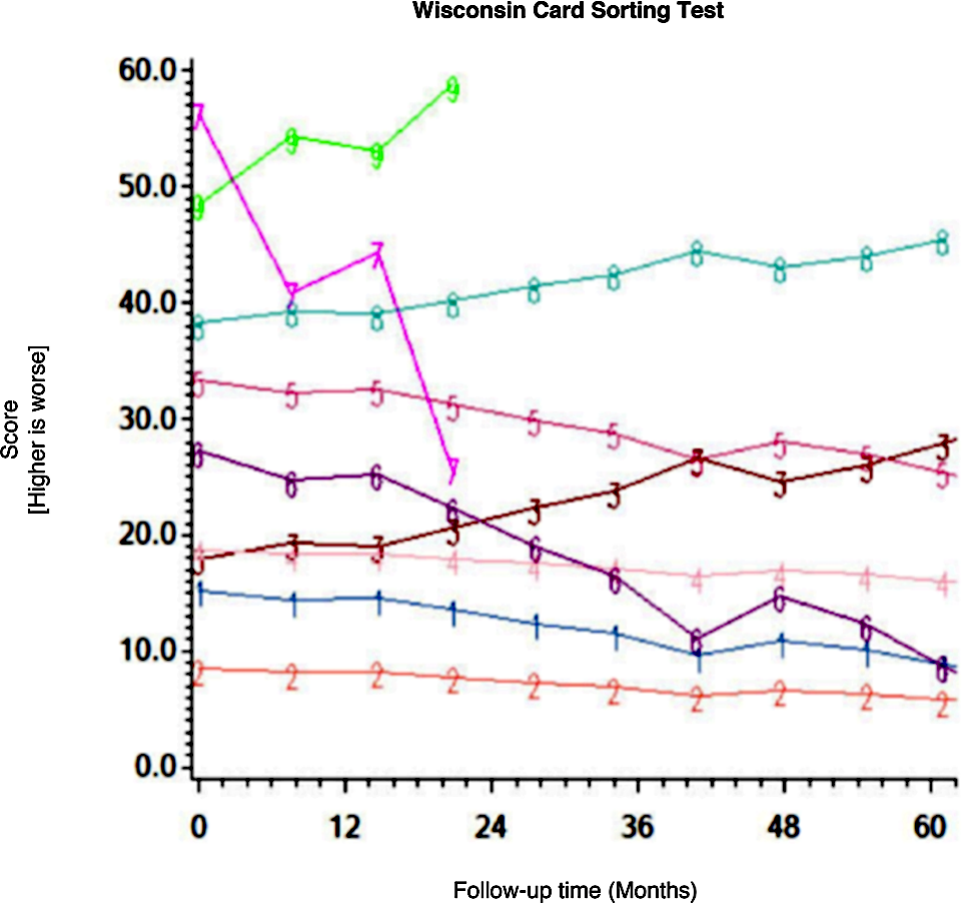
Results of group-based trajectory analysis for Wisconsin Card Sorting Test.

**Fig 6:**
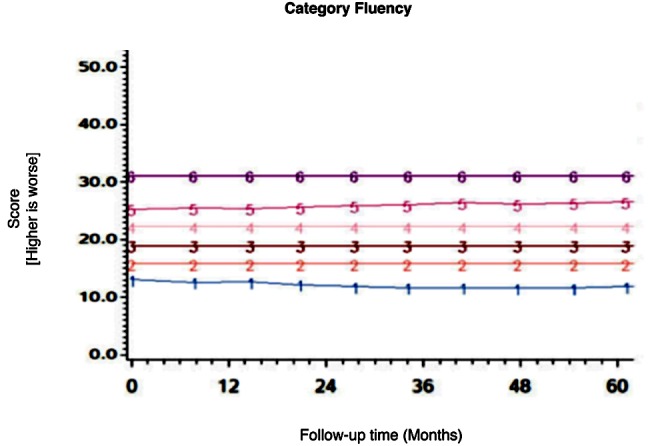
Results of group-based trajectory analysis for Category Fluency.

**Fig 7:**
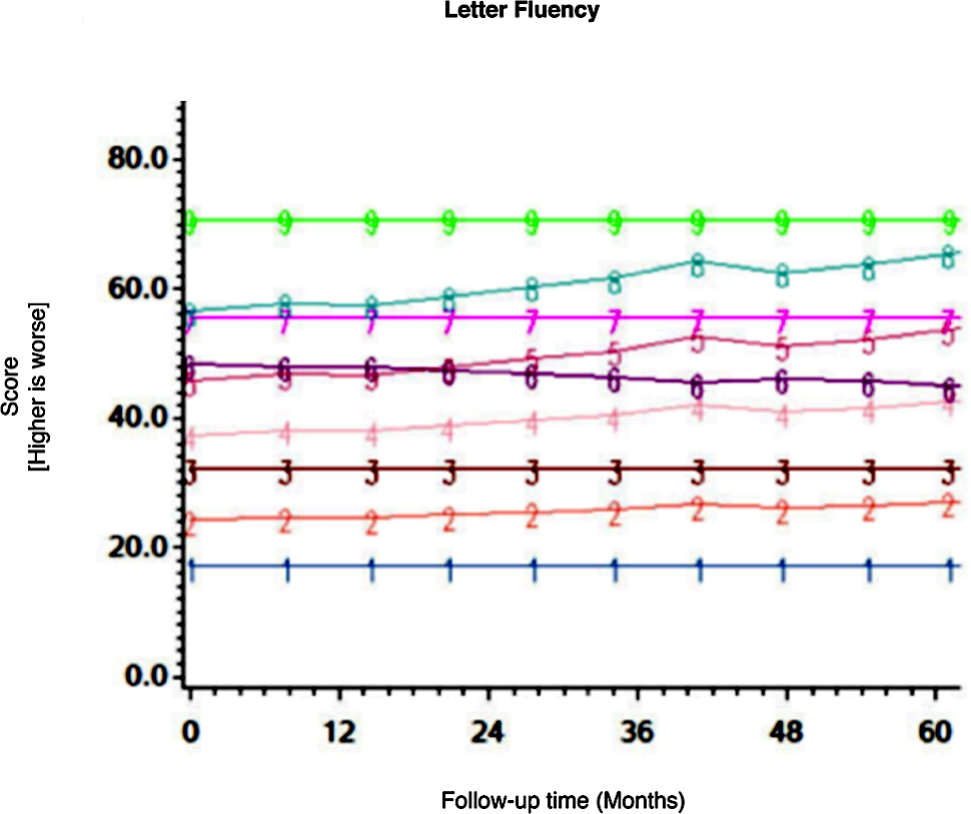
Results of group-based trajectory analysis for Letter Fluency.

**Fig 8:**
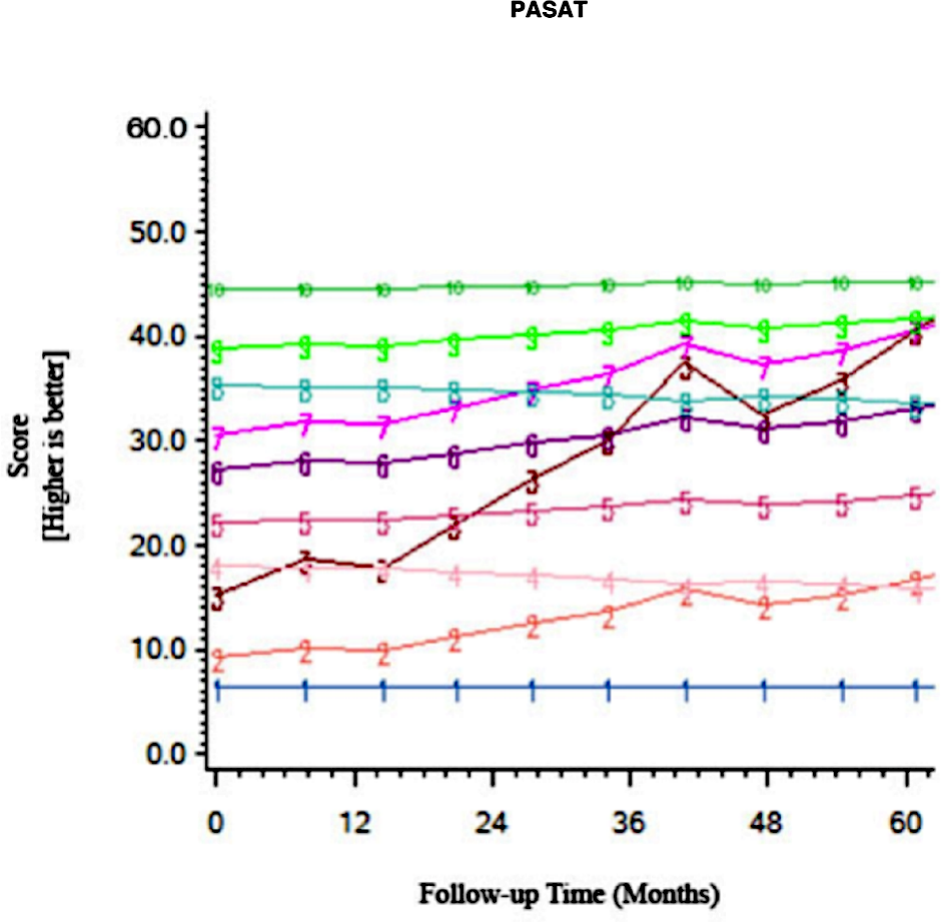
Results of group-based trajectory analysis for Pace Auditory Serial Addition Test (PASAT).

**Fig 9:**
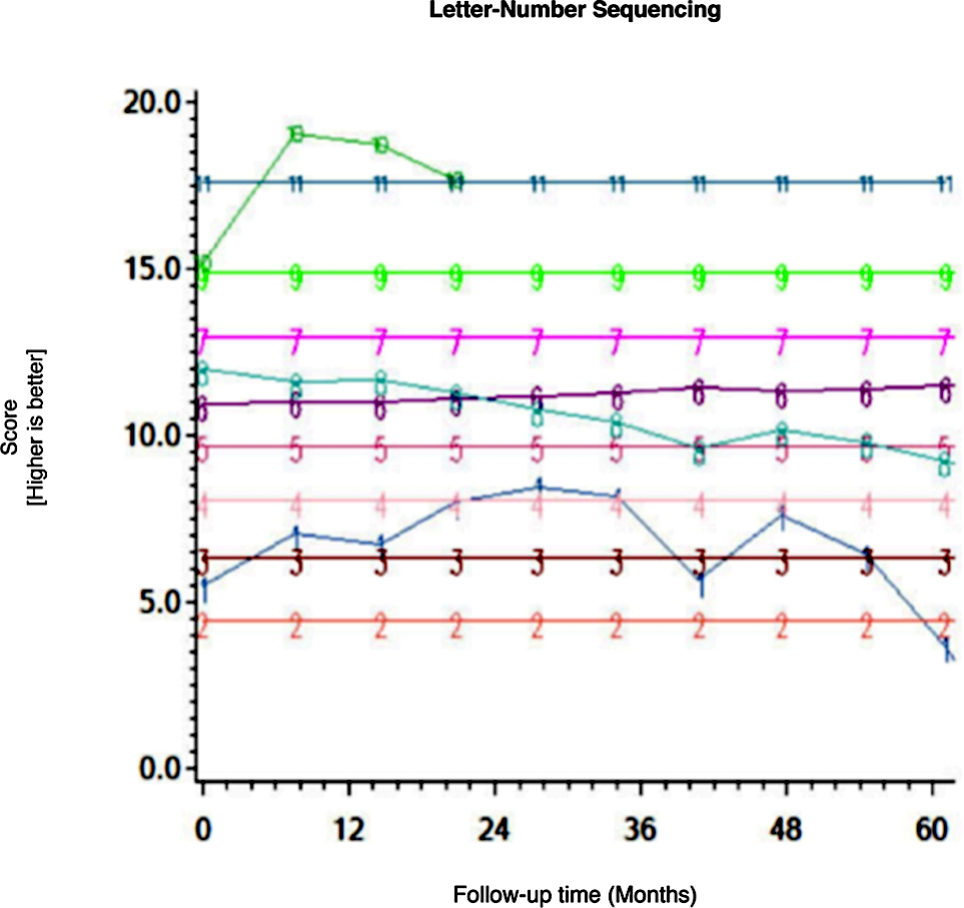
Results of group-based trajectory analysis for Letter-Number Sequencing.

**Fig 10:**
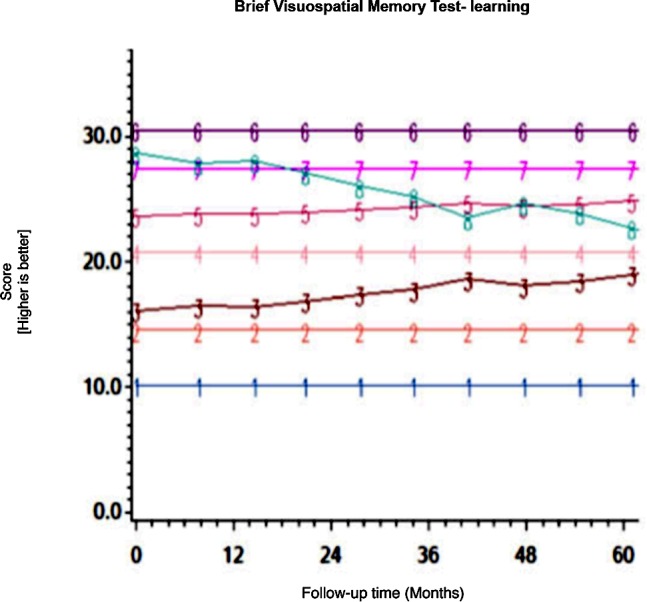
Results of group-based trajectory analysis for Brief Visuospatial Memory Test- Learning.

**Fig 11:**
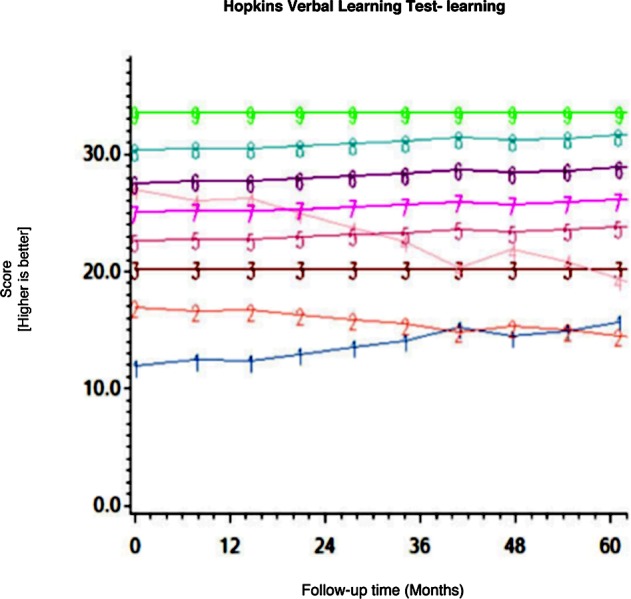
Results of group-based trajectory analysis for Hopkins Verbal Learning Test- Learning.

**Fig 12:**
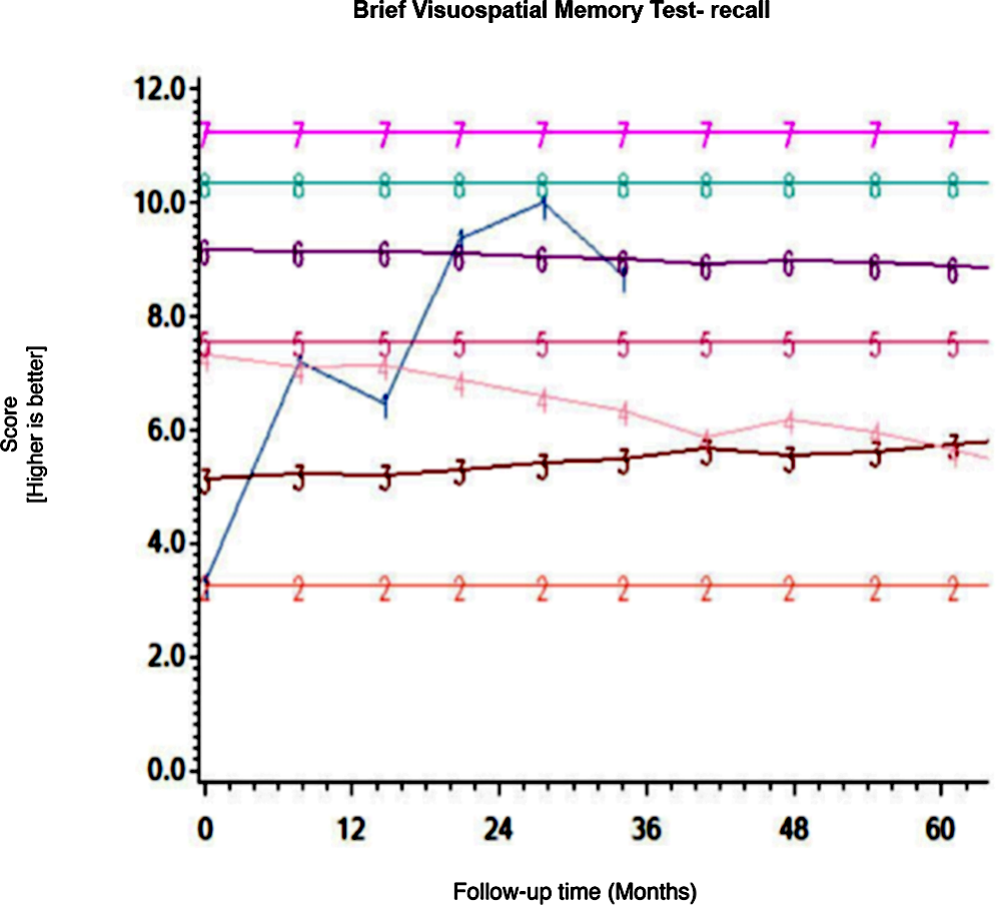
Results of group-based trajectory analysis for Brief Visuospatial Memory Test- Recall.

**Fig 13:**
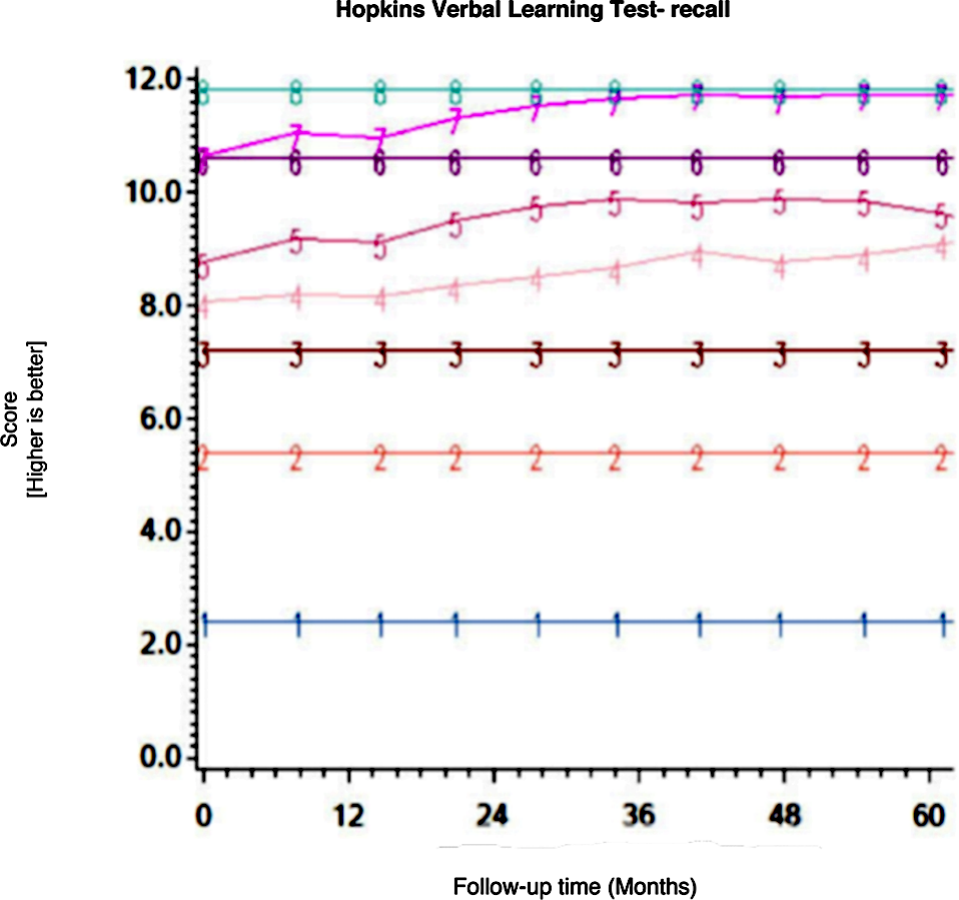
Results of group-based trajectory analysis for Hopkins Verbal Learning Test- Recall.

**Fig 14:**
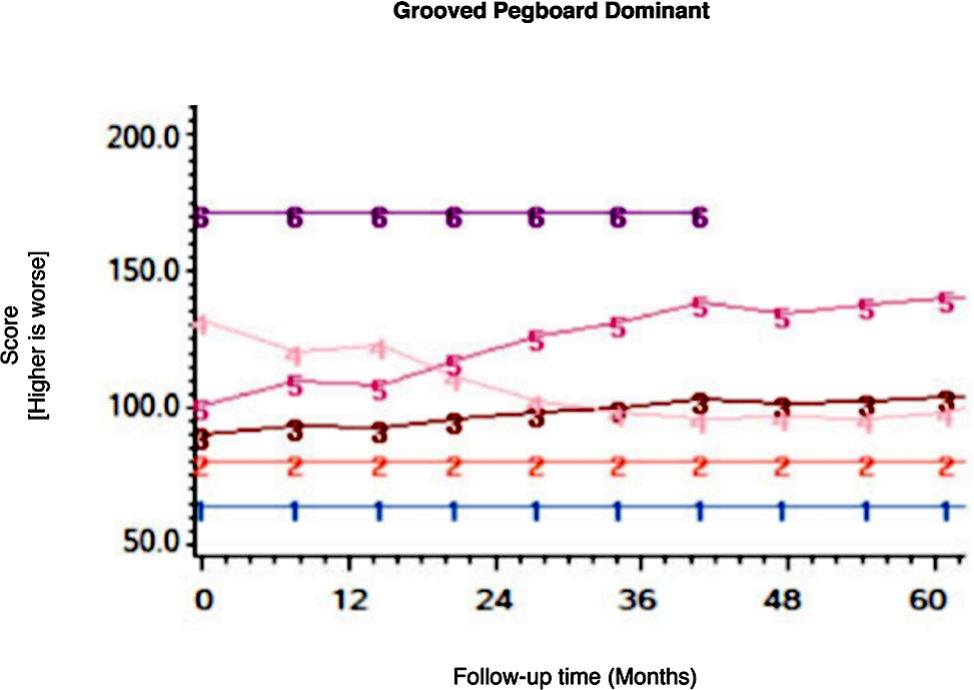
Results of group-based trajectory analysis for Grooved Pegboard- Dominant hand.

**Fig 15:**
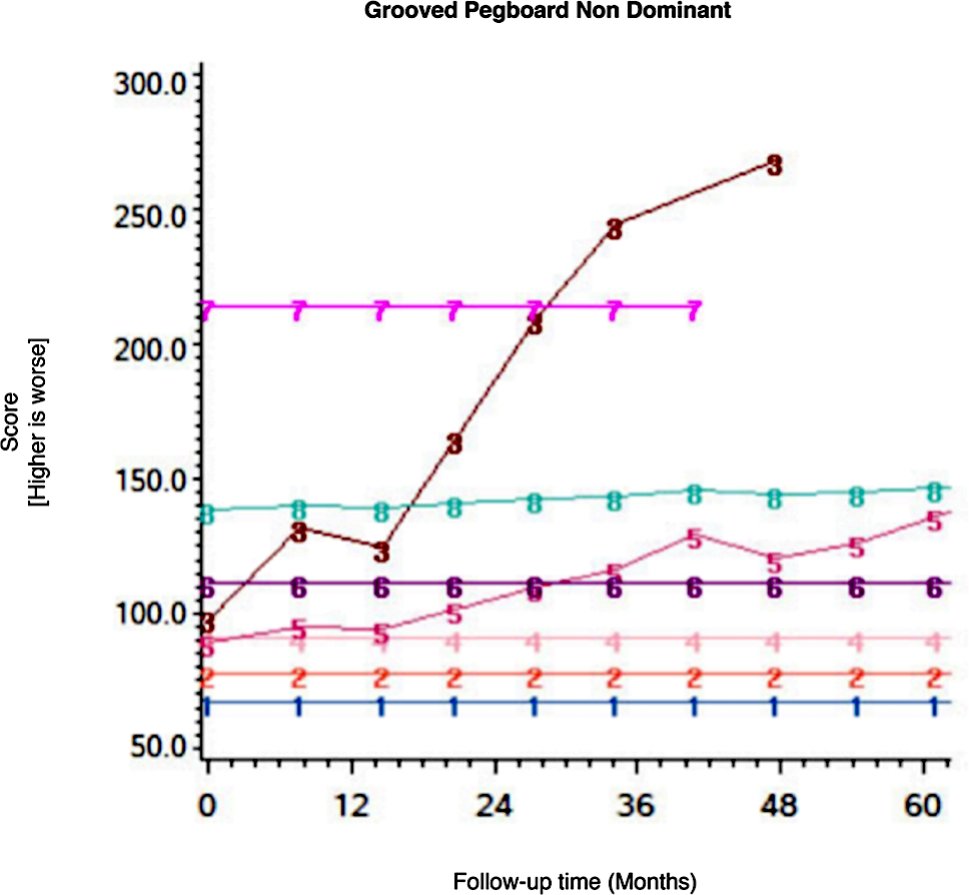
Results of group-based trajectory analysis for Grooved Pegboard- Non-dominant hand.

The posterior probability for group assignment was ≥ 80% for the vast majority of participants with more than 2 testing sessions, and for 71% of those with only 2 sessions. Table 2 shows the proportion of the sample assigned to each trajectory for each test and the classification of the trajectories (i.e. stable, improved or declined). The smallest “groups” were comprised of two individuals (see Letter Number Sequencing and BVMT-Learning), whereas the largest group included 399 participants (see Grooved Pegboard Dominant).

**Table 2:** Proportions of the sample that were assigned to each type of trajectory (i.e. stable, improved or declined) for each test.

	Trajectory number, number (%) assigned to each trajectory, and categorization											
	S/stable; D/declined; I/improved											
Test	1	2	3	4	5	6	7	8	9	10	11	12
TMT-A	285	250	114	3	29	20						
	(41%)	(36%)	(16%)	(<1%)	(4%)	(3%)						
	S	S	S	D	S	I						
Digit Symbol	12	59	55	111	123	97	124	78	31	11		
	(2%)	(8%)	(8%)	(16%)	(18%)	(14%)	(18%)	(11%)	(4%)	(2%)		
	S	S	S	S	S	S	S	S	S	I		
Symbol Search	15	38	67	36	151	127	18	125	71	6	37	10
	(2%)	(5%)	(10%)	(5%)	(22%)	(18%)	(3%)	(18%)	(10%)	(1%)	(5%)	(1%)
	S	S	S	S	S	S	I	S	S	S	S	S
TMT-B	323	243	32	69	19	15						
	(46%)	(35%)	(5%)	(10%)	(2%)	(2%)						
	S	S	D	I	S	I						
WCST	167	360	13	93	14	39	4	7	4			
	(24%)	(51%)	(2%)	(13%)	(2%)	(6%)	(1%)	(1%)	(1%)			
	S	S	D	S	I	I	I	S	D			
Category Fluency	37	170	251	143	79	21						
	(5%)	(25%)	(36%)	(20%)	(11%)	(3%)						
	S	S	S	S	S	S						
Letter Fluency	18	84	124	231	123	35	62	13	11			
	(2%)	(12%)	(18%)	(33%)	(18%)	(5%)	(9%)	(2%)	(1%)			
	S	S	S	S	S	S	S	S	S			
PASAT	5	42	9	72	100	58	96	16	123	180		
	(1%)	(6%)	(1%)	(10%)	(14%)	(8%)	(14%)	(2%)	(18%)	(26%)		
	S	S	I	S	S	S	S	S	S	S		
L-N Sequencing	2	30	56	169	197	143	61	3	29	5	6	
	(<1%)	(4%)	(8%)	(24%)	(28%)	(20%)	(9%)	(<1%)	(4%)	(1%)	(1%)	
	I	S	S	S	S	S	S	D	S	I	S	
BVMT Learning	34	59	89	168	110	67	172	2				
	(5%)	(8%)	(13%)	(24%)	(16%)	(10%)	(25%)	(<1%)				
	S	S	S	S	S	S	S	D				
HVLT- Learning	6	26	117	7	120	122	164	115	24			
	(1%)	(4%)	(17%)	(1%)	(17%)	(17%)	(23%)	(16%)	(3%)			
	S	S	S	D	S	S	S	S	S			
BVMT-Recall	4	18	94	7	138	186	41	213				
	(1%)	(3%)	(13%)	(1%)	(20%)	(27%)	(6%)	(30%)				
	I	S	S	S	S	S	S	S				
HVLT- Recall	19	100	96	141	122	136	45	42				
	(3%)	(14%)	(14%)	(20%)	(17%)	(19%)	(6%)	(6%)				
	S	S	S	S	I	S	I	S				
GP Dominant	399	225	43	12	16	6						
	(57%)	(32%)	(6%)	(2%)	(3%)	(1%)						
	S	S	D	I	D	S						
GP Non Dominant	232	189	2	189	14	52	5	18				
	(33%)	(27%)	(<1%)	(27%)	(2%)	(7%)	(1%)	(3%)				
	S	S	D	S	D	S	S	S				

Both definitions of decline agreed on each declining trajectory for every single test; i.e. the two definitions of change that we tested here yielded the same classification of individuals as decliners. Table 3 shows the aggregated classifications of the 701 HIV+ individuals in the 15 GBTA models.

**Table 3:** Classification of the 701 HIV individuals in the 15 group-based trajectory analysis models according to change of ≥ 0.5 SD.

		Declined		Improved		Stable	
		n	(%)	n	(%)	n	(%)
**Speed of Information Processing**							
Trail Making Test-A		3	(0.6)	20	(3)	678	(96.4)
Digit Symbol		-		11	(2)	690	(98)
Symbol Search		-		18	(3)	683	(97)
**Executive Function**							
Trail Making Test-B		32	(5)	84	(12)	585	(83)
Wisconsin Card Sorting Test		17	(2)	57	(8)	627	(89)
**Verbal Fluency**							
Category Fluency		-		-		701	(100)
Letter Fluency		-		-		701	(100)
**Attention/Working Memory**							
PASAT		-		9	(1)	692	(98.7)
Letter Number Sequencing		3	(0.4)	7	(1)	691	(98.6)
**Memory-Learning**							
BVMT Total Learning		2	(0.3)	-		699	(99.7)
HVLT Total Learning		7	(1)	-		694	(99)
**Memory- Recall**							
BVMT Delayed Recall		-		4	(1)	697	(99)
HVLT Delayed Recall		-		167	(24)	534	(76)
**Motor Function**							
Grooved Pegboard-Dominant		59	(8)	12	(2)	630	(90)
Grooved Pegboard-Non-dominant		16	(2)	-		685	(98)

Overall, there were 111 participants (15.8%) who declined on at least one of the 15 NP tests over 36 months. Whereas improvement on more than one test was common (n = 55, 16.4% of the sample), decline on more than one test was rare, occurring in only 18 participants (2.6%). Variable performance with improvement in some tests and decline in others was seen in 44 individuals (6.3%).

Of the 15 NP tests administered, only 5 showed a decline in 1% or more individuals; an additional three tests identified at least one individual who declined. The highest proportion of decliners was observed in the Grooved Pegboard dominant hand (n = 59, 8%), followed by the Trail Making Test-B (n = 32, 5%), with remaining tests identifying 2% or less of decliners. Not a single participant declined on tests of verbal fluency (letter and category) or memory-recall (verbal and non-verbal). All trajectories were stable over the first 36 months in > 80% of cohort participants. A substantial number of individuals (n = 167; 24%) *improved* over time on the HVLT-R delayed recall, despite the fact that alternate versions were used at different time points. Table 4 shows the baseline performance on each NP test and selected clinical variables among HIV participants who declined and those who did not.

**Table 4:** Baseline performance on each NP test and selected clinical variables among HIV participants who declined and those who did not.

	Decline (n = 111)			No Decline (n = 590)			
**Neuropsychological Test**	Median	Mean	SD	Median	Mean	SD	P value
**Higher score indicating better performance**							
BVMT Total Learning	17.0	16.9	6.3	22.5	22.0	6.9	(p<0.0001)
BVMT Delayed Recall	7.0	6.4	2.7	9.0	8.6	2.6	(p<0.0001)
Category Fluency	18.0	18.4	5. 2	19.0	19.9	4.8	(p = 0.0040)
Letter Fluency	34.0	34.7	10.6	38.0	39.2	11.3	(p = 0.0001)
HVLT Total Learning	22.0	22.8	4.6	25.0	25.2	5.0	(p<0.0001)
HVLT Delayed	8.0	7.4	2.6	9.0	8.4	2.6	(p = 0.0002)
PASAT-50	23.0	22.4	11.3	33.0	32.2	12.0	(p<0.0001)
WAIS-III Digit Symbol	58.0	58.3	14.6	73.0	72.9	17.1	(p<0.0001)
WAIS-III Letter Number Sequencing	8.0	8.3	2.9	10.0	9.8	2.8	(p<0.0001)
WAIS-III Symbol Search	21.0	21.8	7.2	28.0	29.1	8.5	(p<0.0001)
**Neuropsychological Test**							
**Lower score indicating better performance**							
Grooved Pegboard—Dominant	87.0	87.5	17.7	69.0	71.4	16.3	(p<0.0001)
Grooved Pegboard—Non-dominant	93.0	96.5	19.8	77.0	80.9	21.7	(p<0.0001)
Trail Making Test-A	37.0	39.5	15.4	28.0	29.9	11.1	(p<0.0001)
Trail Making Test-B	97.0	118.0	64.2	72.0	83.8	47.5	(p<0.0001)
Wisconsin Card Sorting Test	17.0	20.4	12.0	12.0	14.6	9.7	(p<0.0001)
**Other parameters of interest**							
Age	46.0	46.2	6.6	43.0	42.7	8.8	(p<0.0001)
Education (years)	12.0	12.3	2.5	13.0	12.8	2.6	(p = 0.0631)
Duration of HIV infection (years)	11.7	11.1	5.8	8.2	8.4	6.2	(p = 0.0002)
Duration of follow-up (years)	3.2	3.8	2.6	2.1	3.1	2.5	(p = 0.0087)
Baseline GDS	0.3	0.8	0.6	0.3	0.4	0.4	(p<0.0001)
		N	%		N	%	
Male		83	74.8		471	79.8	(p = 0.2300)
Education (>12years)		47	42.3		302	51.2	(p = 0.0873)
Ethnicity (non-white)		72	64.9		326	55.3	(p = 0.0608)

Across all 15 NP tests, individuals who declined had, on average, poorer baseline test scores than those who did not. The range and variability in raw test scores at baseline were, however, similar between the two groups. Those who declined were also, on average, significantly older, had a longer duration of HIV infection, more follow-up sessions, and were significantly more likely to have a baseline GDS ≥ 0.5, the recommended cut-off to identify NP impairment [13]. The decliners also tended to be less educated and be of non-white ethnicity, although these differences did not reach statistical significance.

## Discussion

By applying GBTA to detect neurocognitive changes over time in a clinical cohort of people living with chronic HIV infection, we identified distinct trajectories across 15 different NP tests over the course of follow-up. Declining trajectories over the first 36 months were found in 8 of the 15 NP tests. The same individuals were identified as decliners across the two definitions of meaningful change that we employed. Only 15.8% of this sample declined on one or more NP test in the first 36 months of follow-up, somewhat less than the 22.7% rate of decline reported by Heaton et al. among a subset of this sample (n = 436) using a different definition of change (i.e. based on averaging scores of 15 NP tests, and using a regression model to estimate change) [15]. In the wider HIV literature, neurocognitive change has been calculated by subtracting average Z scores from 2–8 NP tests at follow-up, compared to baseline. These major differences in approach make it difficult to compare rates of decline across studies.

The low proportion of decliners identified in the present study is notable, because GBTA is a particularly sensitive method to detect decline as evidenced by the fact that we were able to identify “groups” composed of only two individual (0.2% of sample) with a unique trajectory. Only 18 individuals (2.6%) declined on more than one test, a number too small to allow additional analyses that might help define the optimal number of NP tests for detecting clinically meaningful decline. However, it is reassuring that the predictors of decline (defined using one NP test) were biologically plausible.

The posterior probability of group assignment was high (≥ 80%) in most participants, even in those with only two test sessions. This indicates that two observations can yield useable information on probable longitudinal change using GBTA, which provides a means of assigning individuals to probable trajectories even with partial data. This is a major strength of GBTA when applied to longitudinal NP data as it can minimize attrition bias. This is especially important in view of the likelihood that individuals who are more cognitively impaired may be more likely to be lost to follow-up [16]. However, while GBTA overcomes some of the challenges posed by longitudinal data by providing a robust approach to identifying trajectories, interpreting the meaning of these trajectories requires additional information.

The variation in proportion of stable, declined and improved trajectories across the 15 tests suggest that certain cognitive domains could be more severely affected in people with HIV. The Grooved Pegboard dominant and non-dominant hand both identified some decliners (respectively, 8 and 2%), indicating that decline in complex motor function could be a sensitive indicator of brain dysfunction. The TMT-B identified the second highest proportion of decliners (5%), but performance on TMT-A was stable or improved in >99%: this suggests that deterioration in executive function, as opposed to psychomotor slowing alone, accounts for this decline. These findings are consistent with several HIV neuroimaging studies and neural investigations that suggest a diffuse cortico-subcortical process, with psychomotor slowing and impairment in executive skills being most affected [17]. The NP tests in cognitive domains that are more sensitive to regional cortical function, such as declarative memory, did not identify any decliners in this cohort. This is in contrast to the pattern expected in Alzheimer’s disease, where declarative memory is affected early. An alternative explanation for the different rates of decline on various NP test is differences in the distribution of scores on particular tests. Tests with a large range of possible raw scores and a distribution that approximates normal are more suitable to detect change[4].

Our data on NP decline differs from that on cross-sectional impairment at study entry reported by Heaton et al. [18]. Whereas Grooved Pegboard showed most of the decline in our analysis, baseline impairment on that test was found in only 35% of those with neurocognitive impairment (NCI); similarly, very few cohort participants showed decline on memory (learning and recall) whereas impairment in learning was seen in > 60% of cohort participants with NCI at study entry. This contrast between cross-sectional and longitudinal patterns of impairment may reflect the different phenotypes between the “legacy effect” of longstanding, untreated HIV infection versus the ongoing CNS injury which is known to occur in spite of adequate treatment and may be less driven by HIV itself. Supporting this hypothesis is the fact that CHARTER participants, at study entry, had a mean duration of HIV infection > 9 years, a mean nadir CD4 cell count < 200 and among those on cART (71%), only 56% were virologically suppressed [1], thereby exposing the CNS to the ongoing deleterious consequences of viral presence.

Our approach to the identification of cognitive decline is novel and departs from traditional analytic methods that have been predominantly used in the neuropsychology literature. The most notable difference is that it relies exclusively on data from the study sample; no norms were applied to determine either an expected performance or trajectory over time. To quote George E. P. Box, “Essentially, all models are wrong, but some are useful” [19]. We can ask whether the current model is useful. In the clinical setting, NP data are interpreted by an expert who carefully considers the suitability of the normative data in the interpretation of the test scores. This nuanced interpretation is typically not possible in the research setting, where strict cut-offs are applied. The suitability of normative samples is an ongoing source of criticism in the field of neuroHIV where the population may often differ in many, potentially unmeasurable ways (cultural, lifestyle, etc) from normative samples [20]. The norm-free approach we take here avoid this problem, but has its own drawbacks, in that the data are not contrasted against an “expected” performance that would inform its interpretation. Here, over 80% of this cohort had stable performance over time on all tests; thus, over a 36-month time period, stability in cognition is the “expected” pattern. This statement is based on empirical data from a large sub-sample of the cohort, which would seem an appropriate comparison group for our purposes.

GBTA overcomes some of the challenges posed by longitudinal data, providing a robust approach to identifying trajectories. However, interpreting the meaning of these trajectories requires additional information. We tested two definitions of GBTA-based neurocognitive decline here, and found that they yielded identical results in classifying individuals as decliners. We can provide some internal evidence of validity in that, at baseline, decliners defined by these criteria were more impaired on every NP test than non-decliners, and were more likely to be classified as having NP impairment according to standard research criteria (GDS ≥ 0.5). This raises the possibility that decliners were already on a declining trajectory at the time they entered the cohort. Decline was also associated with known personal risk factors for HIV-Associated Neurocognitive Disorder such as older age and longer duration of HIV infection [21–23]. However, whether these criteria for change correspond to a clinically meaningful change, or identify a change that might shed light on underlying mechanisms, has yet to be established. These are empirical questions that will require converging evidence from biological, clinical and health outcomes research.

In summary, the present study identified distinct trajectories in each of the 15 NP tests over 36 months using GBTA. Heterogeneity in trajectories was also noted across tests. Delineation of such heterogeneity is key to the identification of risk factors for decline, a first step in the development of interventions aimed at decreasing incident cognitive morbidity. It is reassuring to see that, in spite of the high rates of neurocognitive impairment reported in several cross-sectional studies [1,24–27], neurocognitive *decline* was observed in only a small subset of this study cohort, and that decline was on a single test in most cases. This work shows a novel approach to analyzing longitudinal NP data that has analytic advantages over simpler methods. Further research is needed to test whether it can shed useful light on the underlying mechanisms and the clinical relevance of neurocognitive decline defined in this way.
